# Comparison of Immunoprotective Efficacy of Six Antigenic Proteins of *Pasteurella multocida* Serotype a in KM Mice (*Mus musculus*)

**DOI:** 10.3390/pathogens15060580

**Published:** 2026-05-28

**Authors:** Wenjing Zhang, Yiming Guo, Lijun Guan, Lifang Si, Zhanqin Zhao

**Affiliations:** Luoyang Key Laboratory of Animal Bacterial Infectious Disease Prevention and Control Technology, College of Animal Science and Technology, Henan University of Science and Technology, Luoyang 471000, China

**Keywords:** *Pasteurella multocida*, protective antigen, subunit vaccine, immunoprotection

## Abstract

*Pasteurella multocida* serotype A (*P. multocida*) is frequently associated with severe respiratory disease in swine (*Sus scrofa*), highlighting the need for effective preventive strategies. To identify protective antigens suitable for a subunit vaccine targeting porcine *P. multocida* infection, six recombinant proteins (rAspA, rLolA, rOmpP6, rOppA, rRps6, rSmpA) were expressed in a prokaryotic system, and their efficacy was evaluated in a *Mus musculus* (Kunming) mouse model. All proteins were purified using His-tag affinity chromatography, and SDS-PAGE analysis confirmed expression with bands at the expected molecular weights (61, 26, 21, 63, 19, and 17 kDa). Each protein, formulated with ISA 201 adjuvant, was administered to mice in two immunizations. Indirect ELISA of sera collected at multiple time points demonstrated that all vaccines induced high antigen-specific IgG levels. rOppA, rLolA, rOmpP6, and rRps6 were expressed in soluble form, whereas rAspA and rSmpA formed inclusion bodies. In a lethal challenge model, rLolA and rRps6 conferred the highest protection (60% each), followed by rAspA and rOmpP6 (30%), rOppA (20%), and rSmpA (10%). Under the conditions tested, the highest protection observed was 60%, and none of the six antigens achieved complete protection against homologous A7 challenge in mice. This first head-to-head comparison under identical conditions provides a reference framework for future antigen screening studies.

## 1. Introduction

*Pasteurella multocida* (*P. multocida*) is a zoonotic pathogen capable of infecting a broad range of hosts, including domestic animals such as pigs, cattle, sheep, and poultry, as well as pets, wild animals, and humans. It is classified into five capsular serogroups (A, B, D, E, and F) based on capsular antigens, with serogroups A and D being the predominant serotypes in swine (*Sus scrofa*), causing pneumonic pasteurellosis and progressive atrophic rhinitis, respectively [1]. Notably, *P. multocida* serotype A frequently acts as a secondary pathogen in the porcine respiratory disease complex, often coinfecting with immunocompromising viruses such as porcine reproductive and respiratory syndrome virus (PRRSV) and porcine circovirus type 2 (PCV2) [2], as well as other bacterial pathogens including *Streptococcus suis* and *Glaesserella parasuis* [3]. The antigen candidates in this study were evaluated in a pneumonic pasteurellosis model, which is pathologically distinct from the progressive atrophic rhinitis caused by toxigenic strains.

Vaccination remains a key preventive strategy for controlling *P. multocida* serotype A infection [4]. However, traditional inactivated and attenuated live vaccines have limitations, including limited protection, risk of virulence reversion, and inconsistent immune responses. DNA vaccines offer the advantage of immunizing against multiple pathogens and are cost-effective, but their large-scale application is constrained by several drawbacks [5]. Consequently, current research focuses on identifying protein antigens with robust immunogenicity and broad-spectrum protective potential to facilitate the development of novel *P. multocida* subunit vaccines.

Six *P. multocida* proteins were selected as potential subunit vaccine candidates based on previous studies. AspA, a key enzyme in aspartate metabolism, contributes to bacteria’s adaptation to stress [6,7], but its role in immune protection is unclear. Rps6, part of the 30S ribosomal subunit, has a surface-exposed region [8,9]. Given their proven immunogenicity, ribosomal proteins like Rps6 are considered potential immune targets [10], though data on their protective roles are limited. LolA, a conserved outer membrane ABC transporter, shows potential immunogenicity and cross-protection [11], but its protective effect in porcine-derived strains needs further study. The P6-like outer membrane protein is widely distributed across serotypes and is viewed as a potential broad-spectrum antigen [12,13]. OppA, a conserved oligopeptide-binding protein, may serve as a diagnostic antigen and vaccine candidate, but its immunoprotective effect remains unproven. SmpA (BamE) helps assemble outer membrane proteins and maintain membrane stability [14], yet its immunogenicity and protective effects remain unestablished.

Commercial vaccines for porcine *P. multocida* are mainly inactivated bacterins that provide limited cross-protection against heterologous serotypes [15]. Subunit vaccine efforts have focused on conserved surface antigens, such as OmpH and PlpE [4], which show only partial protection in mouse models [16]. The efficacy of other conserved proteins—especially those involved in nutrient uptake (e.g., OppA, LolA) or ribosomal function (e.g., Rps6)—remains underexplored for porcine *P. multocida*. Thus, a systematic comparison of these antigens under identical conditions is needed to identify the best candidates for further development. We constructed, expressed, and purified recombinant plasmids encoding six *P. multocida* antigens in parallel, and then compared their protective efficacy in a mouse immunization and challenge model to identify the most promising candidates for a porcine *P. multocida* subunit vaccine.

## 2. Materials and Methods

### 2.1. Strains and Plasmids

The *P. multocida* A7 serotype A clinical isolate was isolated from the heart blood of a pig in Shandong, China in April 2013, and stored in our laboratory. The expression plasmid pET-28a(+) was constructed and preserved in the laboratory. *Escherichia coli* DH5α and BL21 (DE3) competent cells were purchased from Shanghai Shenggong Bioengineering Co., Ltd. (Shanghai, China). Specific pathogen-free (SPF) female Kunming mice (5–6 weeks old) were purchased from the Experimental Animal Center of Henan Province (Zhengzhou, China).

### 2.2. Main Reagents

Genomic, plasmid, and agarose DNA extraction kits were obtained from Kangwei Century Biotechnology (Taizhou, China) and Beijing Quanshijin Biotechnology (Beijing, China). Restriction enzymes and T4 DNA ligase were provided by Beijing Baori Medical Biotechnology (Beijing, China). The His-tag protein purification kit, kanamycin sulfate (Kan), and isopropyl-β-D-thiogalactoside (IPTG) were purchased from Shanghai Shenggong Biotechnology (Shanghai, China). Sijiqing fetal bovine serum was sourced from Zhejiang Tianhang Biotechnology (Deqing, China). Horseradish peroxidase-labeled sheep anti-pig IgG (IgG-HRP) and sheep anti-mouse IgG (IgG-HRP) were obtained from Proteintech (Wuhan, China). Tryptic Soy Agar (TSA) and Tryptic Soy Broth (TSB) media were acquired from BD (Franklin Lakes, NJ, USA). Montanide TMISA 201VG adjuvant (ISA201) was provided by Shanghai Sepico Special Chemicals (Shanghai, China).

### 2.3. Primer Design

Primer design utilized *P. multocida* HB03 gene sequences obtained from GenBank, specifically *aspA* (CP003328.1, AHE63521.1), *lolA* (AHE64434.1), *ompP6* (AHE63662.1), *oppA* (AHE64803.1), *rps6* (AHE65572.1), and *smpA* (AHE64825.1). These sequences served as the genes of interest for subsequent analyses. Signal peptides and transmembrane regions of the six protein sequences were predicted using the NovoPro online tool (https://www.novopro.cn/tools/, accessed on 20 February 2025). SnapGene software (version 6.0.2) was then employed to design primers for *aspA*, *lolA*, *ompP6*, *oppA*, *rps6*, and *smpA*, ensuring that the amplified fragments excluded signal peptides and transmembrane regions. Primers for *aspA*, *lolA*, *ompP6*, and *oppA* were synthesized by General Biology (Anhui) Co., Ltd. (Anhui, China), while primers for *rps6* and *smpA* were synthesized by Shanghai Shenggong Biological Engineering Co., Ltd. (Shanghai, China) (Table 1).

### 2.4. Construction of Recombinant Plasmid

Genomic DNA was extracted from the *P. multocida* A7 strain using a bacterial genomic DNA extraction kit. The target genes *aspA*, *lolA*, *ompP6*, *oppA*, *rps6*, and *smpA* were amplified by PCR using extracted genomic DNA as the template. Amplicons were verified by 1% agarose gel electrophoresis, and DNA fragments of the expected size were recovered using an agarose gel extraction kit. Purified fragments were cloned into the pMD19-T vector, ligated at 16 °C for 16 h, and transformed into *E.coli* DH5α competent cells. Plasmids were extracted and sequenced. Following sequence verification, each recombinant pMD19-T vector was digested with specific restriction enzymes to recover the gene inserts. These inserts were ligated into the pET-28a(+) expression vector, which had been digested with the same restriction enzymes, and transformed into *E. coli* DH5α competent cells after a 16-h ligation at 16 °C. Recombinant expression plasmids (pET-28a-aspA, pET-28a-lolA, pET-28a-ompP6, pET-28a-oppA, pET-28a-rps6, and pET-28a-smpA) were thus constructed. The recombinant plasmids were then double-digested with restriction enzymes, and the resulting products were sent to Shanghai Shenggong Bioengineering Co., Ltd. for sequencing confirmation.

### 2.5. Induction, Expression and Purification of Recombinant Protein

Verified recombinant plasmids (e.g., pET-28a-AspA) were transformed into *E. coli* BL21 (DE3) competent cells. Transformed cells were plated on LB agar plates containing kanamycin and incubated at 37 °C for 16 h. Single colonies were inoculated into LB medium containing kanamycin and cultured at 37 °C with shaking at 180 rpm for 12 h. The resulting culture was diluted 1:100 into fresh LB medium containing kanamycin and incubated at 37 °C and 180 rpm until the OD 600 nm reached 0.6 to 1.0. IPTG was added to induce expression of recombinant proteins (rAspA, rLolA, rOmpP6, rOppA, rRps6, rSmpA) at 37 °C, 180 rpm for 4 h. Following induction, cells were harvested by centrifugation at 4 °C, 10,000× *g* for 6 min. The pellet was washed twice with pre-cooled PBS, resuspended, sonicated in an ice bath, and centrifuged again at 10,000× *g*, 4 °C for 30 min. The supernatant (soluble fraction) and pellet (inclusion bodies) were collected. The recombinant proteins were designated as rAspA, rLolA, rOmpP6, rOppA, rRps6, and rSmpA.

### 2.6. Immune Protection Test in KM Mice (Mus musculus)

Seventy SPF female KM mice (*Mus musculus*) (5–6 weeks old) were randomly divided into seven groups (*n* = 10) and immunized subcutaneously with PBS plus ISA 201 adjuvant (control) or the respective recombinant protein emulsified with ISA 201 adjuvant at a 1:1 mass ratio (250 µg/mL), with a booster given on day 21. Blood was collected from seven mice per group on days 0, 21, and 35, of which six randomly selected samples per group were used for ELISA analysis. Sera were separated (3500 rpm, 5 min) and stored at −20 °C. For challenge, the *P. multocida* A7 strain was cultured on TSA with 5% fetal bovine serum, and then in TSB with 0.5% fetal bovine serum and 0.1% defibrinated sheep blood (37 °C, 15 h, followed by 1:100 dilution and 12 h incubation), and the challenge dose was adjusted to 210 colony-forming units (CFU)/0.2 mL based on preliminary plate counts (LD50 = 52 CFU, Reed–Muench method). Serum antigen-specific IgG titers were measured by indirect ELISA (100 ng/well coating, sera from 1:20, HRP-conjugated goat anti-mouse IgG at 1:5000, TMB, 450 nm). At 14 days post-booster, mice were challenged intraperitoneally with ~4 LD50 (210 CFU) and monitored daily for 14 days, with severe lethargy or labored breathing as humane endpoints; survivors were euthanized at day 14, and bacterial re-isolation was performed from lung and heart tissues on TSA blood agar.

### 2.7. Statistical Analysis

Statistical analyses were conducted using GraphPad Prism version 9.5.1. Antibody titer data underwent log10 transformation to approximate a normal distribution. Antibody titers were compared using a two-way analysis of variance (ANOVA) with treatment group and time point as factors, followed by Bonferroni’s post hoc test for multiple comparisons. A *p* value less than 0.05 was considered statistically significant. Data are reported as mean with 95% confidence intervals where indicated.

## 3. Results

### 3.1. Construction and Identification of Recombinant Plasmid

**Figure 1:**
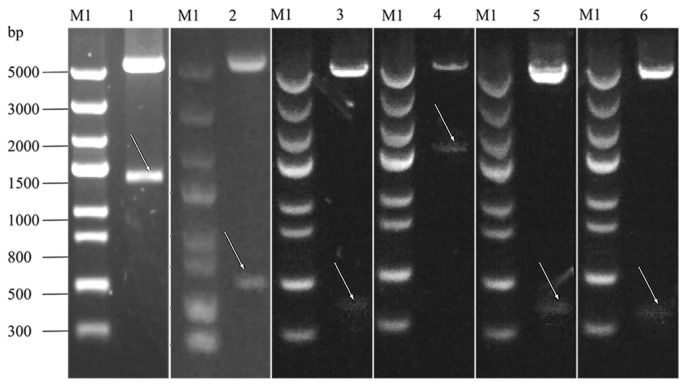
Identification of recombinant expression plasmids using double enzyme digestion. M1: DNA Marker DL5000. Lanes 1-6: double enzyme digestion products of recombinant plasmids pET-28a-AspA, pET-28a-LolA, pET-28a-OmpP6, pET-28a-OppA, pET-28a-Rps6, and pET-28a-SmpA. Arrows indicate the target gene fragments (*aspA*, *lolA*, *ompP6*, *oppA*, *rps6*, and *smpA*).

The target genes were amplified from *P. multocida* A7 genomic DNA using specific primers. PCR and double enzyme digestion confirmed that the *aspA* (1416 bp), *lolA* (549 bp), *ompP6* (393 bp), *oppA* (1560 bp), *rps6* (378 bp), and *smpA* (357 bp) fragments, as well as the linearized pET-28a(+) vector (~5.3 kb), corresponded to the expected sizes (Figure 1). Sequencing analysis verified the accuracy of each gene. The recombinant plasmids pET-28a-AspA, pET-28a-LolA, pET-28a-OmpP6, pET-28a-OppA, pET-28a-Rps6, and pET-28a-SmpA were successfully constructed.

### 3.2. SDS-PAGE Analysis of Recombinant Protein Expression and Purification

**Figure 2:**
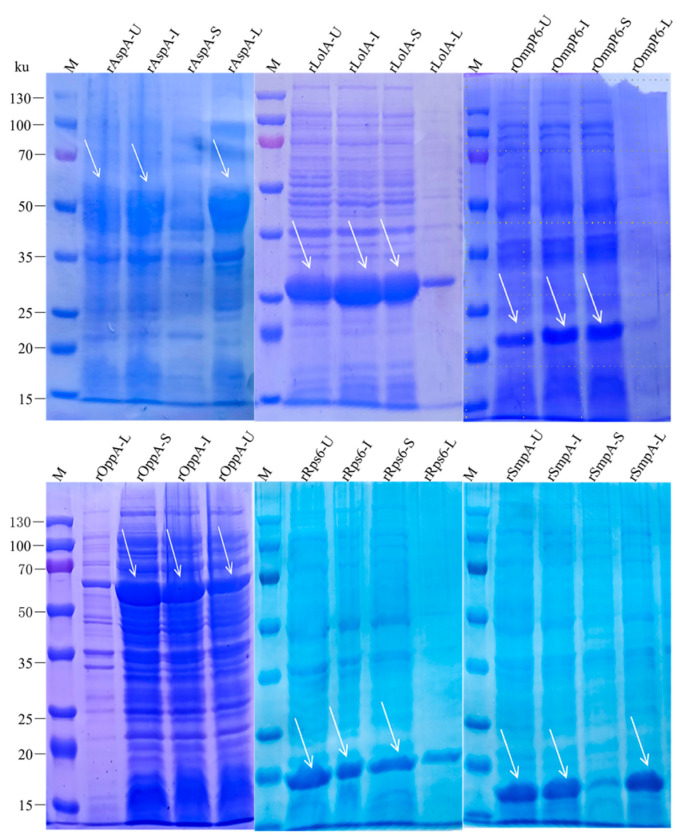
SDS-PAGE analysis of recombinant protein expression. M: Protein marker; rAspA-U and rAspA-I: uninduced and induced cultures of BL21/pET28a-AspA, respectively; rAspA-S and rAspA-L: supernatant and precipitate of induced BL21/pET28a-AspA following ultrasonication. Other proteins are labeled similarly. Arrows indicate the positions of each recombinant protein.

**Figure 3:**
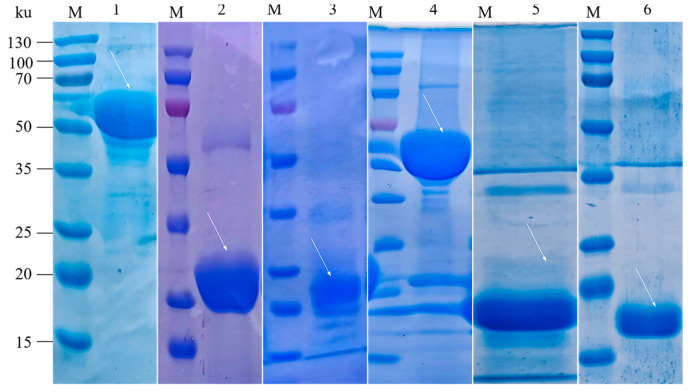
SDS-PAGE analysis of purified recombinant proteins. M: protein marker; lanes 1–6: purified rAspA, rLolA, rOmpP6, rOppA, rRps6, and rSmpA.

SDS-PAGE analysis confirmed the expression of all six recombinant proteins, with molecular weights of 61, 26, 21, 63, 19, and 17 kDa for rAspA, rLolA, rOmpP6, rOppA, rRps6, and rSmpA, respectively. rLolA, rOmpP6, rOppA, and rRps6 were detected in the supernatant, indicating soluble expression, while rAspA and rSmpA were present in the pellet as inclusion bodies (Figure 2). After purification using a His-tag protein purification kit, target bands corresponding to the expected sizes were observed for all six recombinant proteins (Figure 3). The concentrations of these proteins, determined with a micro-volume nucleic acid protein analyzer, were 1.5 mg/mL, 8.0 mg/mL, 1.0 mg/mL, 1.2 mg/mL, 3.0 mg/mL, and 2.5 mg/mL, respectively.

### 3.3. Indirect ELISA for Detection of Antibody Levels in Mice

**Figure 4:**
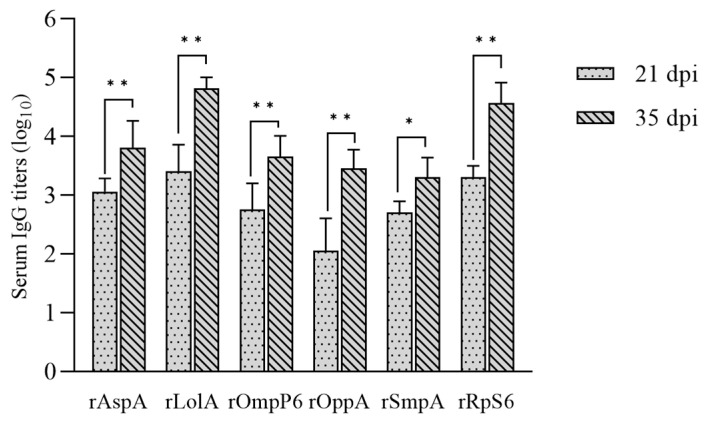
Serum antigen-specific IgG antibody titers in mice immunized with six recombinant *P. multocida* proteins. Data are presented as mean log10 titers of six mice per group, with error bars representing the 95% confidence interval. * *p* < 0.05; ** *p* < 0.01.

Serum antibody levels were quantified using indirect ELISA on days 0, 21, and 35. After the second immunization, the rAspA, rLolA, rOmpP6, rOppA, and rRps6 groups exhibited highly significant increases relative to their post-prime levels (*p* < 0.01), whereas the rSmpA group showed a significant but smaller increase (*p* < 0.05) (Figure 4). After two immunizations, the rLolA group exhibited the highest geometric mean antibody titer (1:65536), followed by rRps6 (1:36781), rAspA (1:6502), rOmpP6 (1:4598), rOppA (1:2896), and rSmpA (1:2048). These findings suggest that the protein vaccines effectively stimulate humoral immune responses, as evidenced by increasing specific antibody titers over time.

### 3.4. Results of the Immune Protection Test in Mice

Fourteen days after booster immunization, each group of ten mice was challenged intraperitoneally with approximately 4 LD50 of the *P. multocida* A7 strain. Mice in the PBS negative control group began to exhibit symptoms 12 h post-challenge, and all died within three days. Observed symptoms included depression, ruffled fur, reduced mobility, lack of response to external stimuli, purulent eye discharge, shortness of breath, and acute, sudden death. Necropsy of deceased mice revealed lung swelling and hemorrhage, splenomegaly, and liver hemorrhage. *P. multocida* was isolated from the heart, lungs, liver, and other tissues of these mice. Recombinant protein vaccine groups demonstrated varying degrees of morbidity and mortality. At the conclusion of the 14-day observation period, necropsy of all surviving mice revealed no significant pathological changes in any organs, and no *P. multocida* was isolated from heart or lung tissues. The rLolA and rRps6 protein vaccine groups exhibited the highest immune protection, with a 60% (6/10) survival rate. The rAspA, rOmpP6, and rOppA protein vaccine groups showed protection rates of 30% (3/10), 30% (3/10), and 20% (2/10), respectively, indicating partial protection. The rSmpA protein vaccine group demonstrated the lowest immune protection, with a 10% (1/10) survival rate (Table 2).

## 4. Discussion

*P.multocida* is one of the bacterial pathogens frequently isolated from pigs with respiratory disease in China, and bacterial infections including those caused by *P. multocida* represent a considerable economic burden for the swine industry [3]. These outcomes underscore the urgent need for enhanced prevention and control strategies. Subunit vaccines represent a promising solution. In the present study, all six recombinant *P. multocida* antigens significantly increased antigen-specific IgG antibody levels in mice, demonstrating robust immunostimulatory activity [17].

For protein expression, the six recombinant proteins were induced at 37 °C for 4 h. Under these conditions, rOppA was present in both soluble and inclusion body forms, while expression at 16 °C resulted primarily in soluble protein. In contrast, rSmpA and rAspA formed inclusion bodies at 37 °C, and their expression remained predominantly as inclusion bodies even at 16 °C. It should be noted that rAspA and rSmpA were purified from inclusion bodies under denaturing conditions, and the immunogenicity of refolded material may not fully reflect that of the natively folded protein. To enhance protein solubility and facilitate purification, future studies should further optimize expression conditions, including IPTG concentration, induction time, and temperature, to achieve soluble expression of rSmpA and rAspA [18]. Antibody levels are a key indicator of antigen immunogenicity and the capacity to elicit humoral immune responses [19]. Indirect ELISA results showed that all six recombinant protein vaccines effectively induced high titers of antigen-specific IgG antibodies in mice. Among these, rLolA and rRps6 elicited the strongest antibody responses, which was consistent with their superior performance in subsequent challenge experiments.

Among the six antigens evaluated, rLolA and rRps6 exhibited the highest protection among the six antigens tested, each conferring 60% protection against lethal homologous challenge. The protective efficacy of rLolA is consistent with its established immunogenicity across Pasteurellaceae species, including 50% protection against *Glaesserella parasuis* [11] and 10–40% protection against *Actinobacillus pleuropneumoniae* [20]. The high sequence conservation of LolA among *P. multocida* strains likely contributes to this cross-protective potential [21]. For rRps6, the 60% protection observed in this study is lower than the 80% reported by Luo et al. [22] in an avian *P. multocida* model. This discrepancy may be attributable to differences in challenge dose, as the present study used 4 LD50 (210 CFU) compared to 7 CFU per animal in Luo et al., or to host-specific variations in antigen recognition. Despite these differences, the consistent immunogenicity of Rps6 across studies supports its further development as a vaccine component. The remaining antigens demonstrated more modest protective effects. rAspA conferred 30% protection in this study, which is substantially lower than the 80% reported by Luo et al. [22] at a much lower challenge dose (7 CFU per animal). This dose-dependent reduction in protection is similar to the pattern observed for rRps6. OmpP6, evaluated here for the first time in porcine *P. multocida*, also provided 30% protection, indicating moderate potential, while rOppA conferred 20% protection. SmpA yielded the lowest protection (10%), which may be attributable to its expression as an inclusion body. Notably, fusion of SmpA with other antigens has been shown to enhance immunogenicity in *A. baumannii* [23], suggesting a potential strategy to improve its performance in future studies.

Collectively, although all six antigens elicited specific antibody responses, none achieved complete protection. This outcome highlights two fundamental challenges in developing *P. multocida* subunit vaccines. First, *P. multocida* pathogenesis involves a diverse array of virulence factors, including capsular polysaccharides, lipopolysaccharides, adhesins, and the protein antigens evaluated in this study [24]. Immunity directed against a single target may therefore be insufficient for complete protection. Second, mice are highly sensitive to *P. multocida* infection (LD50 = 52 CFU in this model); under such stringent conditions, even partial bacterial clearance may not prevent mortality. These findings suggest that a multi-antigen formulation, rather than a single protein, will likely be necessary to achieve sterilizing immunity against *P. multocida*.

Several limitations of this study should be considered. The challenge was conducted exclusively with the homologous A7 strain (serotype A). Although both LolA and Rps6 are reported to be highly conserved proteins [21,25], sequence conservation does not guarantee cross-protective immunity against heterologous serotypes or genetically distinct field isolates of the same serotype. Serotype D is also prevalent in swine herds, yet its susceptibility to rLolA- or rRps6-mediated protection has not been tested. The mouse model, while indispensable for preliminary antigen screening, does not fully replicate the respiratory tract environment, immune ontogeny, or disease manifestations of the natural porcine host. Evaluation of lead candidates in a porcine challenge model is warranted to further assess their protective efficacy. Furthermore, the single-pathogen model used in this study does not reflect the polymicrobial nature of natural *P. multocida* infections, where the bacterium often acts as a secondary pathogen. The 4 LD50 challenge dose was selected to ensure consistent lethality in the control group, thereby allowing clear discrimination of protective efficacy among the six antigens under stringent conditions. However, this dose may represent a higher exposure level than what occurs in natural infections. In addition, the protection observed against a non-toxigenic strain may not extend to the toxigenic strains that cause progressive atrophic rhinitis. Further studies are needed to evaluate the efficacy of these antigens under coinfection conditions and against toxigenic strains. Finally, the 60% protection achieved by rLolA and rRps6, although statistically significant, may not meet the ≥80% benchmark typically required for commercial swine vaccines. Future research should explore combining these lead antigens in a multi-antigen formulation or evaluating alternative adjuvants and delivery strategies to enhance protective efficacy.

In summary, this direct comparison of six *P. multocida* antigens under standardized experimental conditions identifies rLolA and rRps6 as the most protective antigens against homologous A7 challenge in a mouse model. These results establish a foundation for further evaluation, including assessment of cross-protection against heterologous strains and efficacy testing in the natural swine host.

## Figures and Tables

**Table 1:** The primer sets for PCR in this study.

Gene Name	Primer Sequence (5′—3′)	Amplicon Size	Restriction Site
*aspA*	F:GGATCCATGACAGTAACAAGAAAAGAAGTGG	1416 bp	*BamH* I
	R:GTCGACTTTATTCAACTTCGCTTTATAGGTTGG		*Sal* I
*lolA*	F:GGATCCGATGCAGCAAGCGAATTACAGCAAC	549 bp	*BamH* I
	R:AAGCTTTTTTTGGCGTTGGTCATCGAGTTCT		*Hind* III
*ompP6*	F:GGATCCTGTGGTTCATCTAAAAAAGATGAAAGC	393 bp	*BamH* I
	R:GAATTCGTATGCTAACACAGCACGACG		*EcoR* I
*oppA*	F:GAATTCGCAGAAGTACCCGCGGGC R:CCGCTCGAGTTTGACTAAATAAACATCTTTTAG	1560 bp	*EcoR* I *Xho* I
*rps6*	F:CGCGGATCCATGCGACACTACGAAATCG	378 bp	*BamH* I
	R:CCGCTCGAGCTCTTCAGCATCCTCAAAA		*Xho* I
*smpA*	F:CGCGGATCCTGTTCAACTGTAGATAAGCTTGTG	357 bp	*BamH* I
	R:CCTCGAGTTGCCAAAATTTCCACCAGC		*Xho* I

**Table 2:** Results of immune protection tests for six recombinant protein vaccines in mice.

Vaccine Types	Challenge Dose	Number of Mice	Fatality	Survival Number	Protection Rate
rAspA	4 LD50	10	7	3	30% (3/10)
rLolA	4 LD50	10	4	6	60% (6/10)
rOmpP6	4 LD50	10	7	3	30% (3/10)
rOppA	4 LD50	10	8	2	20% (2/10)
rRps6	4 LD50	10	4	6	60% (6/10)
rSmpA	4 LD50	10	9	1	10% (1/10)
PBS	4 LD50	10	10	0	0 (0/10)

Note. The LD50 of the *P. multocida* A7 strain in mice was determined to be 52 colony-forming units (CFU). Mouse survival during the 14-day post-challenge observation period was considered protective.

## Data Availability

The data presented in this study are available on request from the corresponding author.
